# Role of IGFBP7 in Diabetic Nephropathy: TGF-β1 Induces IGFBP7 via Smad2/4 in Human Renal Proximal Tubular Epithelial Cells

**DOI:** 10.1371/journal.pone.0150897

**Published:** 2016-03-14

**Authors:** Jun Watanabe, Yumi Takiyama, Jun Honjyo, Yuichi Makino, Yukihiro Fujita, Masatoshi Tateno, Masakazu Haneda

**Affiliations:** 1 Division of Metabolism and Biosystemic Science, Department of Medicine, Asahikawa Medical University, Asahikawa, Hokkaido, Japan; 2 Department of Pathology, Asahikawa Medical University, Asahikawa, Hokkaido, Japan; National Cancer Institute, UNITED STATES

## Abstract

Tubular injury is one of the important determinants of progressive renal failure in diabetic nephropathy (DN), and TGF-β1 has been implicated in the pathogenesis of tubulointerstitial disease that characterizes proteinuric renal disease. The aim of this study was to identify novel therapeutic target molecules that play a role in the tubule damage of DN. We used an LC-MS/MS-based proteomic technique and human renal proximal epithelial cells (HRPTECs). Urine samples from Japanese patients with type 2 diabetes (n = 46) were used to quantify the candidate protein. Several proteins in HRPTECs in cultured media were observed to be driven by TGF-β1, one of which was 33-kDa IGFBP7, which is a member of IGFBP family. TGF-β1 up-regulated the expressions of IGFBP7 mRNA and protein in a dose- and time-dependent fashion via Smad2 and 4, but not MAPK pathways in HRPTECs. In addition, the knockdown of IGFBP7 restored the TGF-β1-induced epithelial to mesenchymal transition (EMT). In the immunohistochemical analysis, IGFBP7 was localized to the cytoplasm of tubular cells but not that of glomerular cells in diabetic kidney. Urinary IGFBP7 levels were significantly higher in the patients with macroalbuminuria and were correlated with age (r = 0.308, *p* = 0.037), eGFR (r = −0.376, *p* = 0.01), urinary β_2_-microglobulin (r = 0.385, *p* = 0.008), and urinary N-acetyl-beta-D-glucosaminidase (NAG) (r = 0.502, *p* = 0.000). A multivariate regression analysis identified urinary NAG and age as determinants associated with urinary IGFBP7 levels. In conclusion, our data suggest that TGF-β1 enhances IGFBP7 via Smad2/4 pathways, and that IGFBP7 might be involved in the TGF-β1-induced tubular injury in DN.

## Introduction

Diabetic nephropathy (DN) is now a leading cause of end-stage renal failure, and DN therefore constitutes a major portion of progressive kidney disease. For the past several decades, it has been thought that DN is a primarily glomerular disease, rather than a disease based on tubular interstitial lesions. However, in studies using the renal biopsies of non insulin-dependent diabetic patients with microalbuminuria, approx. one-third of the patients had predominantly typical diabetic glomeulopathy, and another one-third had atypical patterns of injury with absent or mild diabetic glomerular changes associated with severe tubulointerstitial lesions and/or arteriolar hyalinosis and global glomerular sclerosis [1,2]. It is now widely accepted that the deterioration of renal function better correlates with the degree of renal tubular interstitial fibrosis rather than with glomerular lesions [3–5].

A key molecule that has been implicated in the pathogenesis of DN is transforming growth factor-beta one (TGF-β1) [6–9]. We demonstrated that metformin has renoprotective effects against DN by rescuing intracellular hypoxia in renal proximal tubular cells, independent of its glucose-lowering effects [10]. Metformin also inhibits profibrotic plasminogen activator inhibitor 1 (PAI-1) in type 2 diabetic subjects [11,12]. In this context, we have explored the proteomic analysis of the cultured media in which human renal proximal tubular epithelial cells were treated with TGF-β1 and/or metformin in order to identify new pathophysiological molecules which might be involved in the tubular injury in DN. We observed that several proteins in the cultured media of human renal proximal epithelial cells (HRPTECs) were driven by TGF-β1 and repressed by metformin, one of which was 33-kDa insulin like growth factor binding protein 7 (IGFBP7), which is a member of the IGFBP family [13]. In the present study, we investigated the regulation of IGFBP7 expression in HRPTECs and performed a western blot analysis and enzyme-linked immunosorbent assay (ELISA) for IGFBP7 using the urinary samples of type 2 diabetic subjects.

## Materials and Methods

### Materials

Metformin was provided by Dainippon Sumitomo Pharma (Osaka, Japan). D-Glucose was purchased from Wako Pure Chemical Industries (Osaka, Japan), and the chemical inhibitors PD98059, SB203580 and SP600125 were purchased from Cell Signaling Technology (Beverly, MA). Recombinant human IGFBP7 was purchased from R&D Systems (Minneapolis, MN). Anti-human IGFBP7 antibodies were purchased from R&D Systems (Minneapolis, MN) and Novus Biotechnologicals (Littleton, CO). Rabbit polyclonal anti-human antibodies for phospho-p44/42 MAPK(Erk1/2)(Thr202/Tyr204), phospho-MAPKAPK-2(Thr334), MAPKAPK-2, phosphor-c-Jun(Ser73) and c-Jun were purchased from Cell Signaling Technology. Anti-human TGF-β1 antibody was obtained from Santa Cruz Biotechnology (Santa Cruz, CA). A mouse monocolonal anti-human CD-15 antibody and a rabbit polyclonal anti-vimentin antibody were purchased from Santa Cruz Biotechnology (Dallas, TX). A mouse monoclonal anti- ZO-1antibody was purchased from BD Biosciences (San Diego, CA). Alexa Fluor 488 donkey anti-rabbit IgG, and Alexa Fluor 594 anti-goat, andt anti-mouse IgG were purchased from Invitrogen (Carlsbad, CA). Smad2 (L-003561), Smad3 (L-20067), Smad4 (L-003902), MAPK1(L-003555), MAPK8(L-003514) and control (non-targeting pool, D-001810) small interference RNAs (siRNAs) were purchased from Dharmacon (Lafayette, CO). IGFBP7 siRNA was purchased from Santa Cruz Biosystems. The sequences of siRNAs which are available from Dharmacon are presented in S1 Table. Other hormones, chemicals and antibodies were obtained from Sigma-Aldrich (St. Louis, MO), unless otherwise indicated.

### Patients

The total of 46 Japanese patients with type 2 diabetes were recruited individuals who regularly visited the outpatient clinic of the Department of Medicine at Asahikawa Medical University (24 males, 22 females, age 61.0 ± 13 yr, mean ± SD). All diabetic patients were defined according to the criteria of the Japan Diabetes Society [14]. Patients who had complicating cancer, liver disease, or nondiabetic kidney disease were excluded from this study. Diabetic nephropathy was classified by a urinary albumin creatinine ratio (normoalbuminuria <30 mg/gCr, microalbuminuria 30–300 mg/gCr, macroalbuminuria >300 mg/gCr). The study was conducted in accordance with the ethical principle of the Declaration of Helsinki and approved by Asahikawa Medical University Research Ethics Committee. We obtained the written informed consent from each patient.

### Tissue samples

Renal biopsy specimens were obtained with the informed consent of the patients with DN, according to the regulations of the ethics committee. The diagnosis of DN was confirmed by histopathological evaluation using light microscopy, immunofluorescence staining and electron microscopy in order to exclude the coexistence of nondiabetic renal diseases. The samples were immediately snap-frozen in liquid nitrogen and stored at −80°C until use.

### Immunohistochemistry

We performed double immunofluorescence with IGFBP7 (goat polyclonal anti-human IGFBP7 antibody, 1:100; R&D systems) and TGF-β1(rabbit polyclonal anti-human TGF-β1 antibody, 1:100; Santa Cruz Biotechnology) using human kidney tissue derived from subjects with type 2 diabetes and overt nephropathy. To further identify the individual nephron segments which were positive for IGFBP7 immunostaining, we performed immunohistochemical analysis using mouse monoclonal anti-human CD-15 antibody (1:50; Santa Cruz Biotechnology) and rabbit polyclonal anti-human IGFBP7 antibody (1:50; Novus Biotechnologicals). Positive staining or CD-15 indicated the segment of proximal tubules [15]. 4 μm formalin-fixed sections were deparaffinized with a series of xylene and ethanol. After heat-induced proteolytic epitope retrieval in sodium citrate buffer (10 mmol/l sodium citrate containing 0.05% Tween 20, pH 6.0) using microwave oven, the sections were treated with a reagent for blocking non-specific background staining (Dako Japan, Tokyo). Then, slides were incubated with primary antibodies overnight at 4°C. Secondary antibodies (ie, donkey anti-rabbit (Alexa Fluor 488) and donkey anti-goat and anti-mouse (Alexa Fluor 594) were diluted in antibody diluent with background reducing components (Dako) and incubated for 2 h at room temperature in the shade. The sections were then washed with PBS three times and finally mounted in aqueous medium with DAPI (Vectashield Mounting Medium with DAPI; Vector Laboratories, Burlingame, CA). Stained sections were observed by fluorescence microscopy (BZ-8100; Keyence, Osaka, Japan), and digital images were collected.

### Cell cultures

HRPTECs were purchased as one or twice-passaged tubular cells from Lonza Walkersville (Walkersville, MD). The cells were grown in renal epithelial cell growth medium (REGM, Lonza) on collagen type 1-coated dishes at 37°C in an incubator containing 5% CO_2_ and 95% humidified air as described [16]. The REGM was supplemented with 0.5% fetal bovine serum (FBS), epidermal growth factor (EGF) (10 ng/ml), insulin (5 μg/ml), hydrocortisone (0.5 μg/ml), epinephrine (0.5 μg/ml), triiodothyronine (6.5 μg/ml), transferrin (10 μg/ml), gentamicin (10 μg/ml), and amphotericin-B (50 ng/ml). Fresh growth medium was added to cells every 2 to 3 days until approx. 80% confluence. Cells were subcultured using trypsin-EDTA digestion. After centrifugation at 1000 rpm, the cells were re-suspended in the medium. All experiments were done using cells at passage 7 or below. For growth arresting, the medium was changed to Dulbecco's modified Eagle's medium (DMEM) (Asahi Technoglass Co., Tokyo) with 0.2% bovine albumin (Trace Biosciences NZ, Auckland, New Zealand) for 48 h before the stimulation experiment. All experiments were subsequently done in serum-free conditions.

### Liquid chromatography-mass spectrometry (LC-MS/MS)

The concentrated cultured media of the cells treated with indicated reagents for 24 h were concentrated 40-fold using Amicon Ultra-4 centrifugal filter units (Millipore, Bedford, MA), and subsequently separated by 5%–20% sodium dodecyl sulfate-polyacrylamide gel electrophoresis (SDS-PAGE) under reducing conditions and stained with Coomassie brilliant blue (CBB). Six bands were excised from the gel and then destained with 50 mM NH_4_HCO_3_ in 50% methanol. After drying the gel in 100% acetonitrile (ACN), 2.5 pmol trypsin in 50 μl of 10 mM Tris (pH 8.5) was added, and in-gel digestion was carried out at 37°C for 16 h.

Digested peptides were extracted by treating the gel twice with 0.1% trifluoroacetic acid (TFA) in 50% ACN and finally extracted once more by 0.1% TFA in 80% ACN for complete extraction. A liquid chromatography-mass spectrometry (MS)/MS analysis was carried out at the Medical Proteomics Laboratory (Graduate School of Frontier Sciences, the Institute of Medical Science, the University of Tokyo, Tokyo). The digested peptides were separated by LC and analyzed by MS/MS followed by a database search using MASCOT version 2.0 (Matrix Science, Boston, MA) [17].

### Real-time quantitative reverse transcription-polymerase chain reaction (qRT-PCR)

Total RNA was extracted from cultured cells using the RNeasy plus mini kit (Qiagen, Tokyo) according to the manufacturer's instructions. The total RNA was dissolved in water and quantitated using an ND-1000 Spectrophotometer, Nanodrop (L.M.S. Co., Tokyo). cDNA synthesis was performed with the SuperScript^™^ III First-Strand Synthesis System for qRT-PCR (Invitrogen). Each cDNA sample was analyzed for gene expression by a quantitative real-time PCR using the fluorescent TaqMan 57-nuclease assay on a sequence detection system (Prism 7300, Applied Biosystems, Carlsbad, CA). The TaqMan real-time PCR was performed using 2× TaqMan Master Mix and 20× assay-on-demand TaqMan primers and probes (Applied Biosystems). The analysis was performed with ABI Prism 7300 SDS software (Applied Biosystems).

Unlabeled specific primers were purchased from Applied Biosystems for detecting the human IGFBP7 gene (Assay ID: Hs 00266026_ml) and the human GAPDH gene (Assay ID: Hs 02758991_g1). Assay details including the region of the gene sequence covered by the primers and amplicon length are shown in the homepage of TaqMan^®^ Gene Expression Assays (http://www5.appliedbiosystems.com/tools/alignMap). After an initial 2 min at 50°C and 10 min at 95°C, the samples were cycled 40 times at 95°C for 15 s and 60°C for 1 min. For the quantitative analysis, the cDNA content of each sample was normalized to the levels of GAPDH as the housekeeping gene using the Comparative C_T_ Method.

### Protein extraction and western blot analysis

HRPTECs were washed twice with PBS and then centrifuged at 6,500 rpm for 3 min. Cells were resuspended in 30–50 μl of ice-cold RIPA lysis buffer (50 mM Tris-HCl, 150 mM NaCl, 0.5% sodium deoxycholate, 0.1% sodium dodecyl sulfate (SDS), 1% Nonidet P-40, 0.004% sodium azide) containing 10 mmol/l phenylmethylsulfonylfluoride (PMSF), the protease inhibitor cocktail, and sodium orthvanadate (Santa Cruz Biotechnology) and lysed for 30 min on ice. After removal of the cell debris by centrifugation at 14000 rpm, the protein concentration in the cell lysate was determined by BCA protein assay (ThermoFisher Scientific, Wilmington, DE). Samples were boiled for 5 min and then analyzed on a 10% NuPage Bis-Tris SDS-PAGE gels (Invitrogen) under reducing conditions. After the gel was soaked in transfer buffer, it was blotted onto a Hybond-P PVDF membrane (Amersham Biosciences, Piscataway, NJ) at 30 V for 1 h.

The blot was blocked overnight in PBS containing 5% nonfat dry milk and 0.1% Tween 20, and then incubated overnight in PBS containing 5% nonfat dry milk, 0.1% Tween 20 and antibodies. After washings with PBS containing 0.1% Tween 20, the blot was stained with 1:2000 dilution of peroxidase-conjugated anti-mouse or anti-rabbit second antibody for 2 h. Bands were visualized by the enhanced chemiluminescence (ECL) system according to the manufacturer's instructions (Amersham). Images were acquired using the Adobe^R^ Photoshop program (Adobe Systems, San Jose, CA), and processed using Multi Guage (Fuji Film, Tokyo) for the densitometric analysis. Signal intensities in control lanes were arbitrarily assigned a value of 1.00. A representative experiment was independently performed three times.

### siRNA and HRPTEC transfection

Silencing of Smad2, Smad3, Smad4, IGFBP7, MAPK1 or MAPK8 gene expression in HRPTECs was achieved by the siRNA technique that has recently been used to study gene function in mammalian cells. The transfection of HRPTECs was carried out by electroporation using the Nucleofection^®^ system (Amaxa, Koln, Germany), according to the protocols proposed by Amaxa. Briefly, 2 × 10^6^ HRPTECs were resuspended in 100 μl of nucleofector solution (Basic Nucleofector Kit for Primary Mammalian Epithelial Cells, Lonza, Allendale, NJ) containing 100 pmol of double-stranded siRNAs (control, Smad2, Smad3, Smad4, IGFBP7, MAPK1 or MAPK8 siRNAs). After electroporation, 500 μl of pre-warmed cultured medium was added to the cuvette, and the cells were transferred into six-well cultures plates containing prewarmed culture medium. Forty-eight hours after transfection, HRPTECs were serum-starved for an additional 24 h and subsequently stimulated with TGF-β1 (2.5 ng/ml) for the indicated times. The expression of the targeted molecule was monitored by Western blotting or a real-time qRT-PCR. To confirm the efficacy of the reduction in the specific RNA on cells transfected with specific RNA relative the control siRNA, we performed qRT-PCR using HRPTECs transfected with IGFBP7 siRNAs, and showed the data in S1 Fig.

### Immunocytochemistry

IGFBP7 knockdown HRPTECs were cultured on four-chamber glass slides (BD Biosciences, San Diego, CA) to reach 80% confluence. After exposure to 2.5 ng/ml TGF-β1 overnight, the cells were fixed with 100% ethanol for 10 min and then incubated with rabbit polyclonal anti-vimentin antibody (1:100; Santa Cruz Biotechnology) and mouse monoclonal anti-ZO-1antibody (1:100; BD Biosciences) at 4°C overnight. The cells were then rinsed in PBS and subsequently incubated with donkey anti-rabbit (Alexa Fluor 488) and donkey anti-mouse (Alexa Fluor 594) secondary antibodies (Invitrogen) at 1:200 dilution overnight at 4°C. Finally, the slides were analyzed by confocal laser-scanning microscopy.

### Measurement of urinary IGFBP7

The urinary concentrations of IGFBP7 were measured using a sandwich enzyme immunoassay kit (USCN Life Science, Houston, TX) in accord with the manufacturer’s instructions. The sensitivity of this assay is >3.47 ng/ml. The intra- and interassay coefficients of variation were <10% and <12%, respectively.

### Statistical analysis

Three separate experiments were performed per protocol, and each treatment group was assayed in triplicate. Values shown represent means ± SD or medians (interquartile range [IQR]). We performed an analysis of variance (ANOVA) and used post hoc Bonferroni tests. The Spearman correlation coefficient was used to analyze the association between two variables. A multivariate regression model with a stepwise forward method was applied to evaluate the independence of factors, using logarithmic transformed values of non-normally distributed variables. All data were analyzed using SPSS (version 17; SPSS, Chicago, IL). *P*-values <0.05 were considered significant.

## Results

### Expression of IGFBP7 in HRPTECs

**Fig 1:**
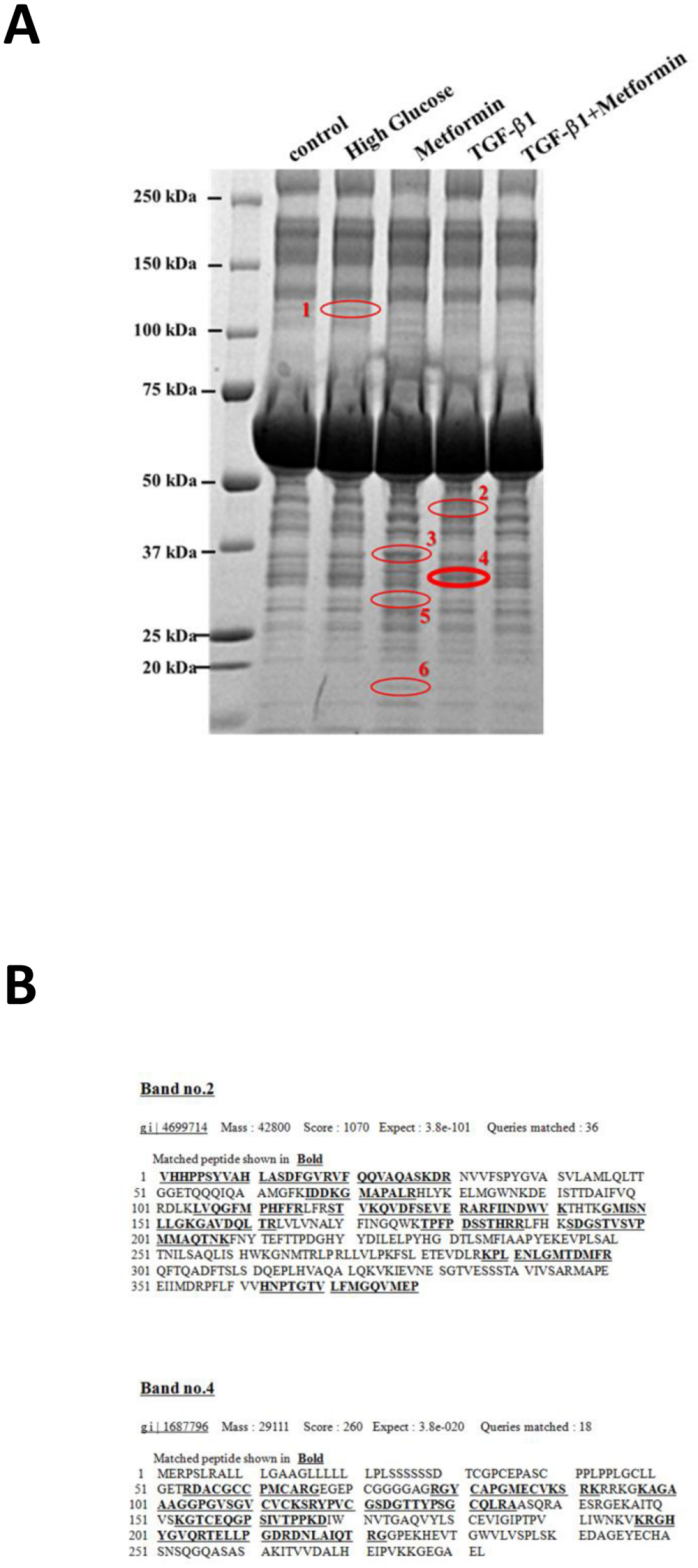
**(A) IGFBP7 was secreted from HRPTECs exposed to TGF-**β**1 using an LC-MS/MS-based proteomic technique.** HRPTECs were treated with D-glucose (30 mM), TGF-β1 (2.5 ng/ml) and/or metformin (10 mM) for 24 h. Samples were separated by 5%–20% SDS-PAGE and detected by Coomassie brilliant blue (CBB). Six bands were excised from the gel, and the digested peptides were separated by LC and analyzed by MS/MS as described in Materials and Methods. **(B) LC-MS/MS, followed by a database search using MASCOT v.2.0 (Matrix Science).** Identified peptides are shown in bold.

To identify novel molecules secreted from human renal tubular cells regulated by TGF- β1 and/or metformin, we used an LC-MS/MS-based proteomic technique. Several proteins were revealed to be regulated by both regents (Fig 1A, Table 1). Two of these bands, which were induced by TGF-β1 and suppressed by metformin, were digested in SDS-PAGE gels by trypsin, and peptide identification was carried out using the LC-MS/MS analysis, followed by a database search with MASCOT ver. 2.0. The proteins for these bands were identified as Plasminogen Activator Inhibitor Type-1(PAI-1) and IGFBP7 (Fig 1B) [13]. High glucose (30 mM) also enhanced the secretion of IGFBP7 (Fig 1A). Because the inhibitory effect of metformin on PAI-1 production has been already known [11,12], we selected IGFBP7 for further studies.

**Table 1:** LC-MS/MS of samples from the cultured media of HRPTECs and the results by a database search using MASCOT.

Band number	Protein	Accession number	Mass	Mascot score	Fold change	Sequence coverage (%)
1	Chain B, Human Complement Component C3	GI:78101268	112869	592	1.1	27
2	Chain A, Human Plasminogen Activator Inhibitor Type-1 in Complex with a Pentapeptide	GI:4699714	42800	1070	25.7	39
3	Chain Q, Crystal Structure Of Human Liver Gapdh	GI:67464046	36312	609	2.2	48
4	Insulin-like Growth Factor Binding Protein 7 [Homo sapiens]	GI:16877961	29111	260	1.3	35
5	Chain A, 14-3-3 Protein Epsilon (human) Complexed to Peptide	GI:67464424	26740	206	3.2	39
6	Peptidylprolyl isomerase A isoform 2 [Homo sapiens]	GI:45439313	11411	198	6.7	46

**Fig 2:**
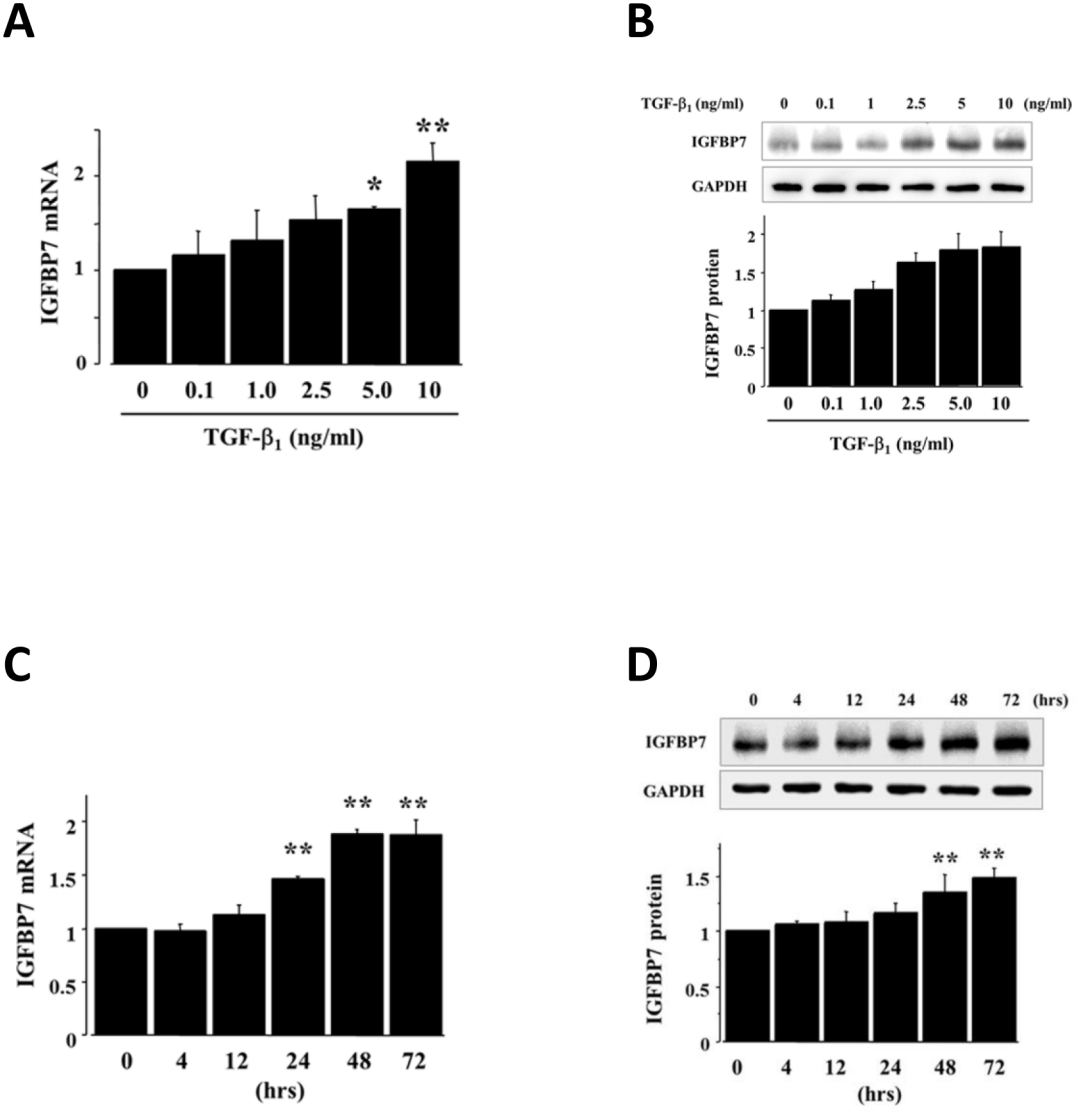
TGF-β1 induced IGFBP7 expression in HRPTECs. HRPTECs were incubated for 48 h with the indicated concentrations of TGF-β1 in serum-free medium. IGFBP7 mRNA expression was determined by real-time RT-PCR **(A)**. The expression of IGFBP7 was normalized to that of GAPDH in the same cell extracts. The expression of IGFBP7 protein in the cell lysates was evaluated by a Western blot analysis **(B)**. The amounts of IGFBP7 were analyzed by western blotting. GAPDH was used as a loading control. The representative immunoblot shows the expression of IGFBP7. TGF-β1 dose-dependently enhanced the expressions of IGFBP7 mRNA **(A)** and protein **(B)** in the HRPTECs. The treatment of HRPTECs for the indicate times with 2.5 ng/ml TGF-β1 showed a time-dependent increase of IGFBP7 mRNA **(C)** and protein expression **(D)**. Each bar represents means ± SD of three separate experiments. *p<0.05 vs. control, **p<0.01 vs. control.

The proteomic data of the TGF-β1-induced IGFBP7 expression in HRPTECs were validated on real-time RT-PCR and western blots (Fig 2). We treated the cells with 0.1 to 10 ng/ml concentrations of TGF-β1 for 48 h (Fig 2A and 2B). The results of the RT-PCR and Western blotting analysis indicated that TGF-β1 stimulated the IGFBP7 expression in a dose-dependent manner. Next, HRPTECs were incubated with TGF-β1 (2.5 ng/ml) for the indicated times from 4 to 72 h, and then we performed a real-time RT-PCR and Western blot analysis for IGFBP7 and GAPDH. TGF-β1 enhanced the IGFBP7 mRNA and protein in a time-dependent fashion (Fig 2C and 2D).

### TGF-β1-induced IGFBP7 expression via the Smad2/4 pathway

**Fig 3:**
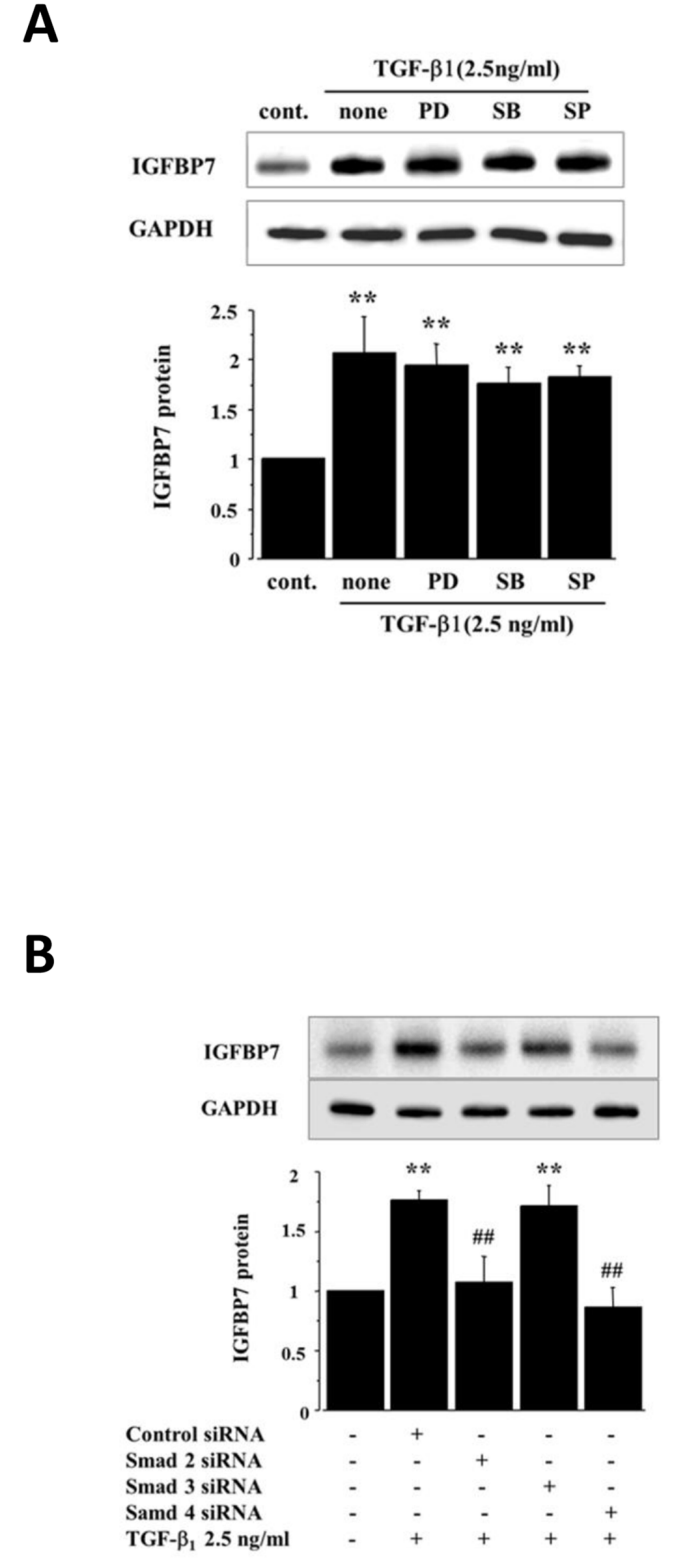
IGFBP7 enhancement by TGF-β1 is dependent on Smad pathways. **(A)** The effects of pretreatment of HRPTECs for 1 h with the Erk1/Erk2 inhibitor PD98059 (PD, 10 μM), the p38 MAPK inhibitor SB203580 (SB, 10 μM), or the JNK inhibitor SP600125 (SP, 10 μM), followed by the administration of TGF-β1 (2.5 ng/ml) for 48 h on the expression of IGFBP7 protein. “None” indicates no administration of the kinase inhibitor. These MAPK inhibitors did not affect the TGF-β1-induced IGFBP7 expression. **(B)** HRPTECs were transiently transfected with control, Smad2-, Smad3-, or Smad4-specific siRNAs (25 nM final concentration). Forty-eight hours after transfection, the HRPTECs were serum-starved for an additional 24 h and then stimulated with TGF-β1 (2.5 ng/ml) for 48 h, and a western blot analysis for IGFBP7 protein was performed. Lowering the expressions of Smad2 and Smad4 proteins reduced the TGF-β1-induced IGFBP7 expression. **p<0.01 vs. control, ^##^*p*<0.01 vs. TGF-β1-treated HRPTECs.

There are two major pathways involved in TGF-β1 signal transduction, the Smad and MAPKs pathways [18–20]. To determine which signal pathway induced by TGF-β1 is critical for stimulating IGFBP7 expression, we first examined the effects of specific inhibitors for MAPKs on the IGFBP7 expression in HRPTECs treated with TGF-β1. We confirmed these inhibitors inhibited the MAPK pathways during the whole experiments for 48h, by examining the expressions of phospho-ERK1/2, phospho-MAPKAPK-2(Thr334) and phospho-c-Jun (Ser73) on HRPTECs treated by the MAPK inhibitors (S2 Fig). The blockades of extracellular signal-regulated kinase (Erk) 1/2 by PD98059, p38 mitogen-activated protein kinase (p38 MAPK) by SB203580, and c-Jun N-terminal kinase (JNK) by SP600125 failed to suppress the TGF-β1 effect on IGFBP7 expression (Fig 3A). In addition, we found that TGF-β1-induced IGFBP7 expressions were not changed by lowering of the expressions of MAPK1 (also known as ERK, p38) and MAPK8(also known as JNK) by siRNA (S3 Fig).

Next, we transfected HRPTECs with control, Smad2-, Smad3-, or Smad4-specific siRNAs (25 nM final concentrations) and then stimulated the cells with TGF-β1 (2.5 ng/ml, 48 h). Lowering the expressions of Smad2 and Smad4 proteins reduced the TGF-β1-induced IGFBP7 expression (Fig 3B). These data indicated that the Smad2/4-pathway was involved in the regulation of the IGFBP7 expression induced by TGF-β1 in the HRPTECs.

### TGF-β1-induced EMT was restored by IGFBP7 gene knockdown

**Fig 4:**
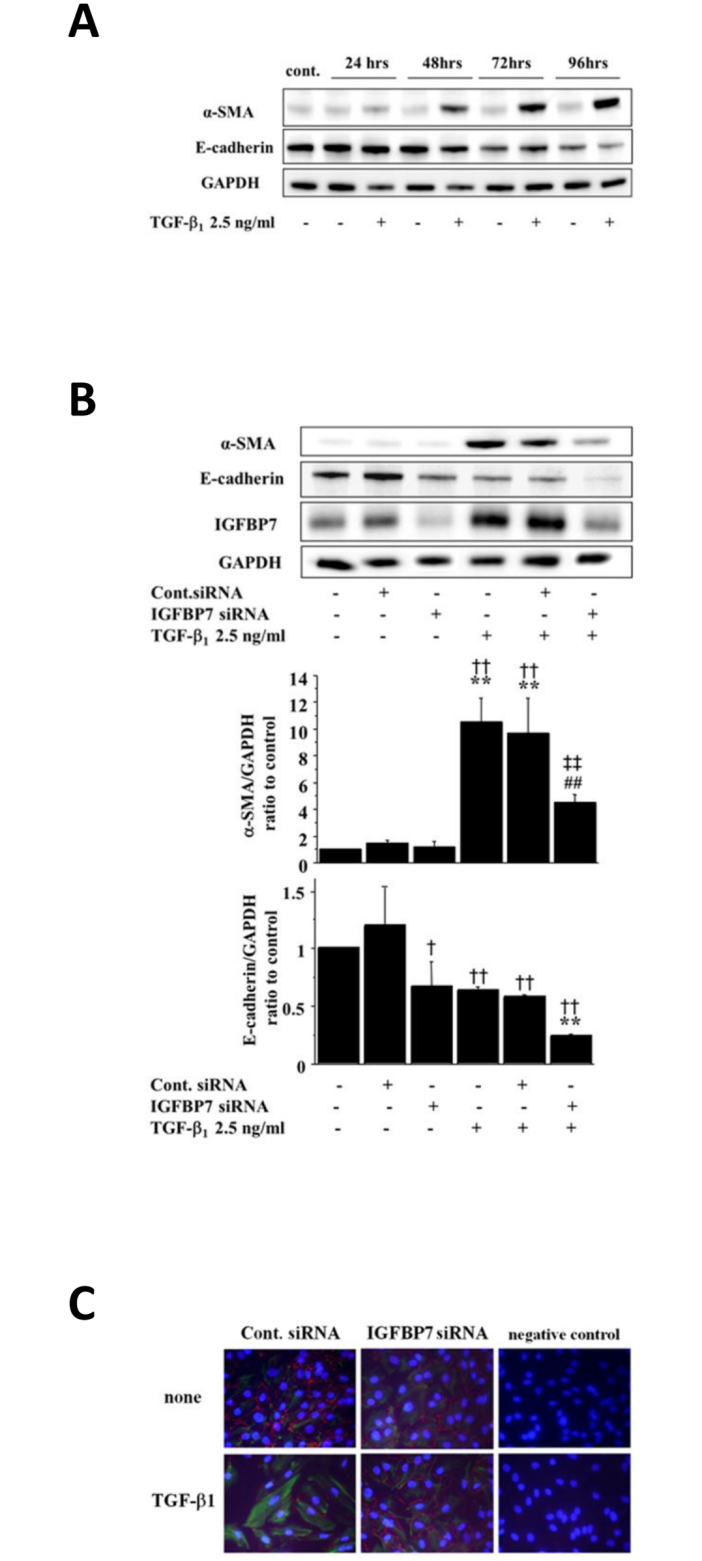
Knockdown of IGFBP7 restored the TGF-β1-induced EMT in HRPTECs. (**A)** TGF-β1-induced an epithelial-mesenchymal-transition (EMT) in HRPTECs. HRPTECs were incubated with 2.5 ng/ml TGF-β1 for the indicated times, and then the extracted proteins were analyzed by western blotting using a mesenchymal marker, α-SMA and an epithelial marker, E-cadherin, respectively. TGF-β1 induced α-SMA expression and decreased the E-cadherin expressions in HRPTECs. (**B)** The knockdown of IGFBP7 attenuated the TGF-β1-induced α-SMA expression in the HRPTECs. HRPTECs were transfected with control or IGFBP7 siRNAs (25 nM final concentrations) and subsequently stimulated with TGF-β1 (2.5 ng/ml, 48 h). Lowering the expressions of IGFBP7 proteins reduced the TGF-β1-induced α-SMA expression, but failed to restore the TGF-β1-decreased E-cadherin expression. Values are means ± SD. **p<0.01 vs. control, ^##^*p*<0.01 vs. TGF-β1-treated HRPTECs, ^†^*p*<0.05, ^††^*p*<0.01 vs. control siRNA-transfected HRPTECs, ^‡^*p*<0.01 vs. control siRNA-transfected and TGF-β1-treated HRPTECs, n = 3. **(C)** HRPTECs were transfected with control or IGFBP7 siRNAs (25 nM final concentrations) and subsequently stimulated with TGF-β1 (2.5 ng/ml, 48 h). Lowering the expressions of IGFBP7 proteins attenuated the TGF-β1-increased mesenchymal marker vimentin expression and restored the TGF-β1-repressed epithelial marker ZO-1 expression and restored the TGF-β1-induced morphological changes. To confirm the first antibodies work, we performed immunocytochemistry without first antibodies as negative control.

TGF-β1 treatment for 24–96 h resulted in an increase in the expression of alpha smooth muscle actin (α-SMA) protein in a time-dependent manner (Fig 4A). A TGF-β1-induced down-regulation of E-cadherin was observed after 96 h of TGF-β1 treatment (Fig 4A).

To determine whether IGFBP7 is involved in the tubular EMT, we transfected HRPTECs with control or IGFBP7-specific siRNA and subsequently stimulated them with TGF-β1 (2.5 ng/ml, 48 h). Lowering the expressions of IGFBP7 protein reduced the TGF-β1-induced α-SMA expression, but it failed to restore the TGF-β1-decreased E-cadherin expression (Fig 4B).

To confirm the effects of IGFBP7 knockdown on the TGF-β1-induced EMT, we performed a Western blot analysis of IGFBP7-knockdown HRPTECs stimulated by TGF-β1, using other antibodies such as vimentin as a mesenchymal marker and ZO-1 as an epithelial marker. The knockdown of IGFBP7 attenuated the TGF-β1-increased vimentin expression and restored the TGF-β1-repressed ZO-1 expression (Fig 4C).

### Immunohistochemical analysis of IGFBP7 in diabetic kidney

**Fig 5:**
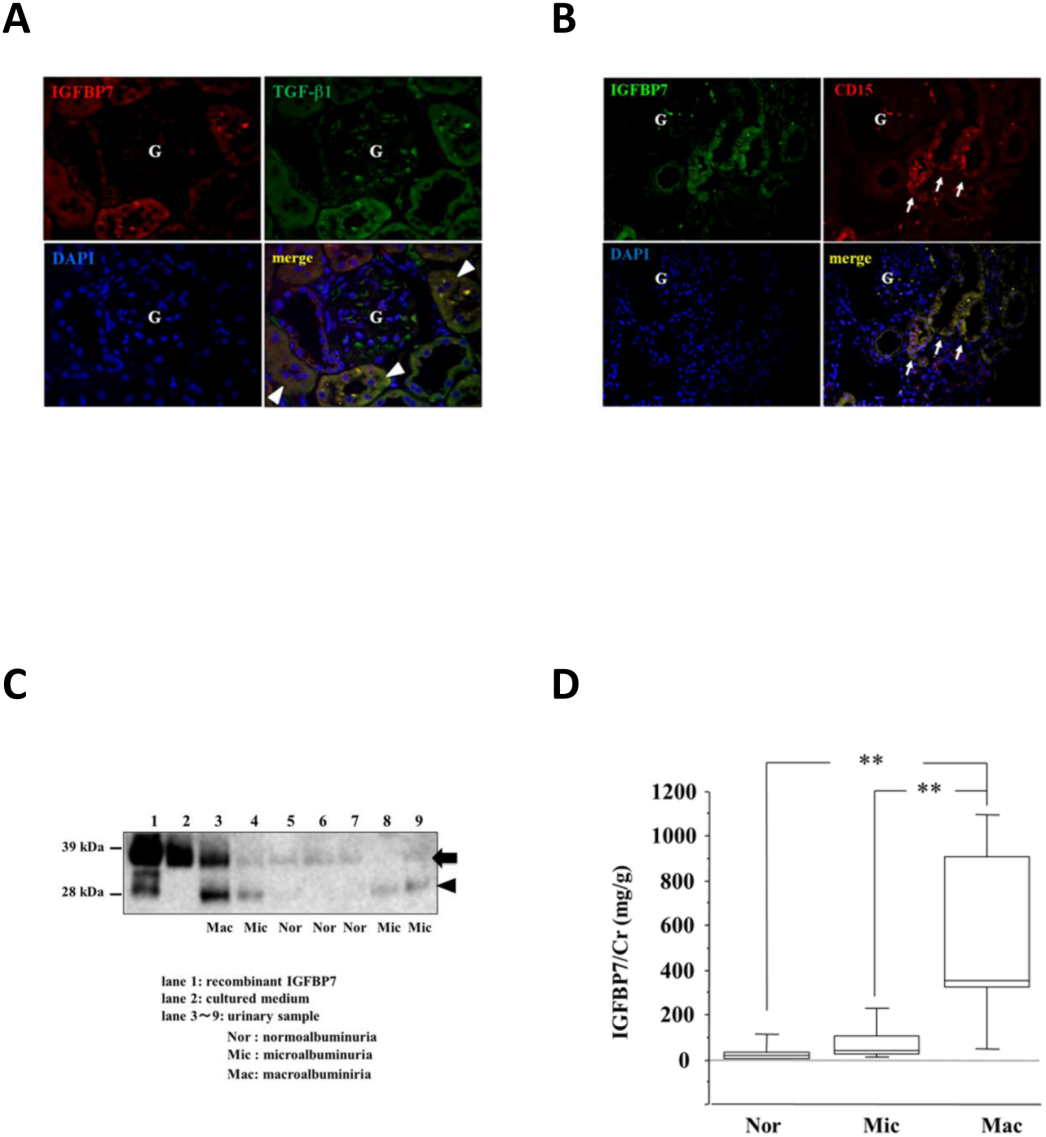
IGFBP7 in human diabetic nephropathy. **(A)** Colocalization of IGFBP7 and TGF-β1 in human renal tubular cells in renal biopsy form type 2 diabetic patients with overt nephropathy. IGFBP7 was localized to the cytoplasm of tubular cells but not that of glomerular cells (G) in diabetic kidney. The confocal microscopy analysis showed the presence of IGFBP7 and TGF-β1 in the proximal tubular cells as indicated by the merged images. The arrowheads indicate the partial colocalization of IGFBP7 and TGF-β1 in proximal tubules. **(B)** IGFBP7 expression in proximal tubular cells. Positive staining for CD-15 indicated the segment of proximal tubules. The proximal tubular cells which are characterized by a brush border and stained by CD15 showed the positive immunostaining for IGFBP7. The arrows indicate the IGFBP7-positive cells observed in CD-15 positive proximal tubules. **(C)** Western blot analysis of IGFBP7 immunoreactive forms in urine from patients with DN or without DN. Urine (10 μl) was processed by SDS-PAGE (10% gels) under reducing conditions and electroblotted onto PDF membranes. Lane 1, recombinant human IGFBP7 (200 ng/lane); lane 2, culture medium (10 μl); lane 3, urine sample from a patient with macroalbuminuria (Mac); lanes 5–7, individual urine samples from patients with normoalbuminuria (Nor); lanes 4, 8 and 9, individual urine samples with microalbuminuria (Mic). Arrow: 33 kDa IGFBP7, arrowhead: 25 kDa cleaved IGFBP7. **(D)** Increased urinary IGFBP7 levels in diabetic nephropathy. The urinary IGFBP7 levels for patients grouped according to different stages of nephropathy as having normoalbunuria (Nor), microalbuminura (Mic), or macroalbuminuria (Mac). Horizontal bars = medians, columns = interquartile ranges, and vertical bars = 95% CIs. *p<0.05, **p<0.01 by one-way ANOVA with a Tukey’s test.

To confirm the localization of IGFBP7 in renal tubular cells, we conducted an immunohistochemical analysis using tissue samples from human kidney in renal biopsies from patients with overt diabetic nephropathy. As shown in Fig 5, the immunohistochemical analysis revealed that IGFBP7 was localized to the cytoplasm of the tubular cells but not glomerular cells in diabetic kidney (Fig 5A) and colocalized with TGF-β1 in the proximal tubules (Fig 5A). In addition, to identify the section of tubules, which were positive for IGFBP7 immunostaining, we performed double immunofluorescence using anti-human CD-15 antibody and IGFBP7 antibody (Fig 5B). IGFBP7-positive cells were observed in CD-15 positive tubular cells (Fig 5B, arrow), indicating that IGFBP7was expressed in proximal tubular epithelial cells.

### IGFBP7 was increased in urine from patients with diabetic nephropathy

We recruited 46 type 2 diabetic patients (24 males) with normoalbuminuria (n = 22), microalbuminuria (n = 16), and macroalbuminuira (n = 8). Table 2 summarizes the clinical characteristics of the patients. The western blot analysis showed the non-cleaved form (33 kDa) and the cleaved form (25 kDa) of IGFBP7 in these urinary samples (Fig 5C). The median level of urinary IGFBP7 in the patients with macroalbuminuria was significantly higher than that in the patients with microalbuminuria and the patients with normoalbuminuria (Fig 5D).

**Table 2:** Clinical characteristics of the study subjects.

n	46
Sex (male/female)	24/22
Age (yrs)	61 ± 13
Duration (yrs)	15 ± 11
BMI (kg/m^2^)	25.3 ± 4.6
HbA1c (%)	7.7 ± 1.6
HbA1c (mmol/mol)	60.9 ± 17.6
Diabetes treatment (diet/oral agents/insulin)	2/22/22
Systolic blood pressure (mmHg)	128 ± 18
Diastolic blood pressure (mmHg)	70 ± 11
Hypertension (%)	65
Taking renin-angiotensin system inhibitors (%)	90
Total cholesterol (mg/dl)	183.8 ± 44.9
Triglycerides (mg/dl)	132(86–184.5)
HDL cholesterol (mg/dl)	50.8 (40.2–61.5)
eGFR (ml/min per 1.73m^2^)	74.9 ± 23.6
eGFR >60	38 (82.6%)
eGFR 30–59	5 (10.9%)
eGFR <30	3 (6.5%)
Albumin:creatinine ratio (mg/g)	38.7 (11.0–176.7)
Normoalbuminuria	22 (48%)
Microalbuminuria	16 (34.7%)
Macroalbuminuria	8 (17.3%)
Urinary β_2_-microglobulin (μg/gCr)	338.6 (106.2–556.6)
Urinary NAG (U/gCr)	7.9 (5.1–17.6)
Urinary IGFBP7 (μg/gCr)	43.8 (24.8–382.4)

Data are mean ± SD for normally distributed variables and median (25th–75th interquartiles) for skewed variables unless otherwise indicated.

The univariate analysis showed that urinary IGFBP7 correlated with renal parameters such as urinary β_2_-microglobulin (r = 0.385, *p* = 0.008), urinary NAG (r = 0.502, *p* = 0.000), and eGFR (r = −0.376, *p* = 0.01) (Table 3). The multivariate regression analysis with a stepwise forward method revealed that the log urinary NAG/Cr (β = 15.7, *p* = 0.001) and age (β = 11.12, *p* = 0.047) are independent determinants of log IGFBP7/Cr (Table 4). In the subjects without metformin treatment (n = 34), the NAG/Cr value was only one factor associated with urinary IGFBP7 (r^2^ = 0.330, β = 0.574, *p* = 0.00). In contrast, in the metformin-treated patients (n = 12), age and HbA1c instead of NAG/Cr were associated with urinary IGFBP7 (r^2^ = 0.748, β = 0.723, −0.397, respectively).

**Table 3:** Factors that correlated with urinary IGFBP7 levels in the univariate analysis.

Parameter	Urinary IGFBP7	
	r	p
Age (yrs)	0.308	0.037
Duration of diabetes (yrs)	0.058	0.703
BMI (kg/m^2^)	−0.254	0.088
HbA1c (%)	−0.184	0.220
Systolic blood pressure (mmHg)	0.000	0.996
Diastolic blood pressure (mmHg)	−0.261	0.079
Total cholesterol (mg/dl)	0.148	0.326
Triglycerides (mg/dl)	0.084	0.579
HDL cholesterol (mg/dl)	0.036	0.811
eGFR (ml/min per 1.73m^2^)	−0.376	0.010
Albumin:creatinine ratio (mg/g)	0.277	0.063
Urinary β2-microglobulin (μg/g Cr)	0.385	0.008
Urinary NAG (U/gCr)	0.502	0.000

Correlations were evaluated with Pearson’s correlation coefficient.

The values of urinary IGFBP7, urinary ACR, urinary β_2_-microglobulin and urinary NAG were log-transformed for the analysis because of their skewed distribution.

ACR, albumin creatinine ration; GFR, glomerular filtration rate.

**Table 4:** Multivariate determinants of log IGFBP7/Cr using a stepwise methodology.

		B	SEM	Standardized β	p-value
**Model 1**	(Constant)	76.6	92.37		
r^2^ = 0.252	NAG/Cr	15.7	4.08	0.52	0.000
**Model 2**	(Constant)	-595.81	340.16		
r^2^ = 0.318	NAG/Cr	14.87	3.96	0.48	0.001
	Age	11.12	5.43	0.26	0.047

## Discussion

The results of this study demonstrated that IGFBP7 was produced and secreted from human renal proximal tubular cells by treatment with TGF-β1. Other nomenclature for IGFBP7 includes menigioma-associated cDNA [21], tumor-derived adhesion factor [22], prostacyclin-stimulating factor [23], angiomodulin [24], and insulin-like growth factor (IGF)-binding protein-related protein 1 [25,26]. Besides these several functions referred to in these studies [21–26], IGFBP7 is distinct from other IGFBPs in that it exhibits a high and specific affinity for insulin [13,27] but a low affinity for IGF [28]. Then, IGFBP7 blocks insulin binding to the insulin receptor by inhibiting the early steps in insulin action [27]. The association between IGFBP7 and type 2 diabetes was reported in two cross-sectional studies that indicated the positive correlation of serum IGFBP7 levels with insulin resistance [25] and endothelium-dependent vasodilation [26]. The present study is the first to report an association between urinary IGFBP7 and diabetic microangiopathy, i.e., DN.

In DN, tubule injury is one of the important determinants of progressive renal failure. TGF-β is well known to be implicated in the pathogenesis of tubulointerstitial disease that characterizes proteinuric renal disease including DN [29,30]. TGF-β plays an important role as a key mediator in renal fibrosis accompanied by the increased expression and accumulation of extracellular matrix (ECM) proteins. Our present findings revealed that IGFBP7 was colocalized with TGF-β1 in the cytoplasm of proximal tubular cells but not the cytoplasm of glomerular cells in diabetic kidney, and that TGF-β1 enhanced the IGFBP7 expression; these results are consistent with those of studies using bovine retinal capillary endothelial cells [31] and hepatic stellate cells (HSCs) [32].

During renal fibrosis, tubular epithelial cells transdifferentiate to mesenchymal cells, which is termed the epithelial-mesenchymal transition (EMT) [33,34]. The phenotypic conversion involves a loss of epithelial polarity and E-cadherin, disruption of the tubular basement membrane, the acquisition of spindle-like morphology, the de novo synthesis of α-SMA, and the production of matrix proteins. Intriguingly, a recent study showed that IGFBP7 has profibrotic activities in vitro [32].

IGFBP7 induces the production of ECM and the development of a myofibroblastic phenotype in HSCs [32]. The inhibitory effect of anti-IGFBP7 antibody on activated HSCs provides a promising intervention for liver fibrosis [32]. However, lowering the expressions of IGFBP7 did not increase the E-cadherin expression in the HRPTECs in our study. The Smad family includes Receptor-regulated (R-) Smads R-Smads (Smad 1,2,3,5 and 8), common (Co-) Smad 4, and inhibitory (I-) Smads 6 and 7 [35]. Among the Smad molecules, Smad2 and Smad 3 play roles as key regulators for TGF-β1/Smad signaling, and they are strongly activated in renal fibrosis in DN [36].

Different roles of Smad2 and Smad3 were shown in TGF-β1 signaling [37,38]. In human proximal tubule epithelial cells, a TGF-β1-induced decrease in E-cadherin was Smad3-dependent, whereas increases in α-SMA expression were dependent on both Smad2 and Smad3 [37]. Therefore, Smad2 and Smad3 are involved in different ways in TGF-β1-induced markers of EMT such as α-SMA and E-cadherin.

Interestingly, in the present study the induction of IGFBP7 by TGF-β1 was dependent on Smad2, and not on Smad3. In addition, IGFBP7 knockdown lowered level of TGF-β1-induced α-SMA, but failed to restore the TGF-β1-decreased level of E-cadherin, which is known to be Smad3-dependent. It is likely that TGF-β1-IGFBP7-EMT signaling is Smad2-dependent and not related to Smad3, and therefore IGFBP7 lowering did not restore the Smad3-dependent TGF-β1-induced decrease in E-cadherin. Moreover, the IGFBP7 lowering augmented the TGF-β1-induced decrease in E-cadherin (Fig 4B), suggesting that Smad2-IGFBP7 signaling might regulate the Smad3-mediated effects on E-cadherin expression.

**Fig 6:**
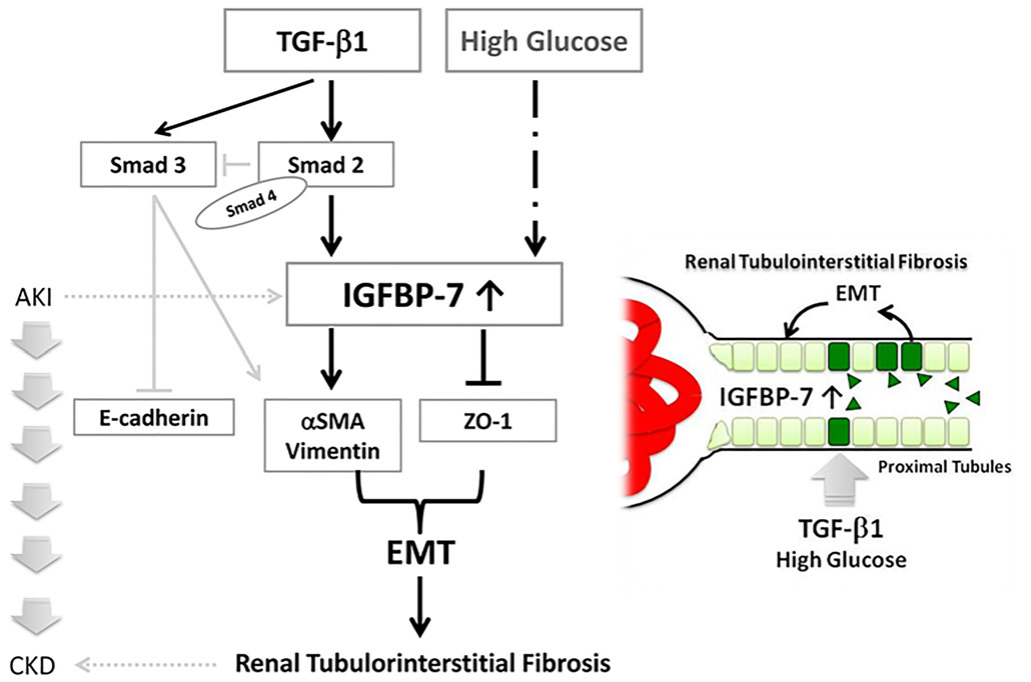
Role of IGFBP7 in Diabetic Nephropathy: TGF-β1 Induces IGFBP7 via Smad2/4 in Human Renal Proximal Tubular Epithelial Cells. Profibrotic TGF-β1 induces IGFBP7 expression in human renal proximal tubular cells via Smad 2/4. IGFBP7 is also involved in the expressions of epithelial marker ZO-1 and mesenchymal markers α-SMA and vimentin, suggesting the potential role of IGFBP7 in epithelial-mesenchymal transition (EMT) leading to renal tubulointersitital fibrosis.

To further test the relationship between IGFBP7 and EMT, we performed immunofluorescense staining using other EMT markers, vimentin and ZO-1. We identified that knockdown of IGFBP7 abolished the TGF-β1-induced vimentin expression, whereas restored TGF-β1 inhibited the ZO-1 expression in the HRPTECs, indicating that IGFBP7 might be involved in the TGF-β1-induced EMT (Fig 6).

Our findings also showed that IGFBP7 was increased in urine from patients with DN. IGFBP7 converted from a single-chain form to a two-chain form (25 kDa and 8 kDa, respectively) by the type II transmembrane serine proteases matriptase [38], which also cleaves the zymogen form of the urokinase-type plasminogen activator (pro-uPA), the inactive proform of the hepatocyte growth factor (pro-HGF), fibronectin, and collagen type IV [39]. The proteolytic cleavage of IGFBP7 results in the loss of its binding activity to insulin and IGF-I and the IGF/insulin-dependent growth-stimulating activity but acquires high syndecan-1-mediated cell adhesion activity [40]. Therefore, the secretion of cleaved type IGFBP7 may be another level in the regulation of IGF/insulin-dependent renal tubular epithelial functions in DN.

We also observed that the urinary IGFBP7 levels were associated with other parameters of renal function, such as eGFR, β_2_-microglobulin, and NAG. However, the urinary IGFBP7 levels did not differ significantly between normoalbuminuria and macroalbuminuria (Fig 5D). On the other hand, the urinary IGFBP7 levels were correlated with age (r = 0.519, *p* = 0.000). Cellular senescence-associated secretory phenotype (SASP) is reported to have crucial roles in age-related conditions [41]. Because IGFBP7 has a central role in BRAFV600E-mediated senescence and apoptosis [42], and because IGFBP7 is also known to be one of the components of SASP [43], aging itself might promote IGFBP7 production. Moreover, IGFBP7 is a urinary marker of cell-cycle arrest in the pathogenesis of acute kidney injury (AKI) [44, 45]. As shown in Fig 1A, we also found that metformin inhibited the TGF-β1-induced IGFBP7 secretion in HRPTECs. It is known that a renin-angiotensin system inhibitor changed the urine proteome pattern in diabetic subjects [46]. Further studies are thus required to assess whether the medication may have acted as a confounder in our study.

Another limitation of the present investigation was that it was a cross-sectional study with a small number of patients and samples, and this precludes determining whether a high IGFBP7 level was a cause or a consequence of DN. However, IGFBP7 was recently shown to be a useful biomarker of acute kidney injury (AKI) even without explanation of the reasons for elevated urinary IGFBP7 levels [44,47–51]. In contrast, an AKI episode increases the risk of advanced CKD in diabetic subjects [52,53]. These studies emphasize that IGFBP7 is a potential candidate for tubular injury in diabetic nephropathy by involvement in EMT leading to AKI-CKD transition. Collectively, our findings suggest that the measurement of urinary IGFBP7 could prove useful in the elucidation of tubular injury and the TGF-β1-induced EMT in DN.

## Supporting Information

S1 Fig Knock-down of the expression of IGFBP7 by specific siRNAs. Forty-eight hours after HRPTECs were transfected with IGFBP7 siRNAs, HRPTECs were serum-starved for an additional 24 h and subsequently stimulated with TGF-β1 (2.5 ng/ml) for 48 h. The efiiciency of knock-down of IGFBP7 by specific IGFBP7 siRNAs was evaluated by qRT-PCR. TGF-β1 significantly induced IGFBP7 mRNA (2.075±0.02, p<0.001). IGFBP7 siRNAs decreased IGFBP7 mRNA expression under 10% of control (without TGF-β1, 0.084±0.02, p<0.001, with TGF-β1, 0.096±0.02, p<0.0001). Three separate experiments were performed per protocol, and each treatment group was assayed in duplicate. Values shown represent means ± SD. We performed an analysis of variance (ANOVA) and used post hoc Bonferroni tests. p**<0.01, p***<0.001, p****<0.0001.
(TIF)

S2 Fig The inhibitory effects of MAPK inhibitors on TGF-β1-stiumulated MAPK pathways. HRPTECs were treated for 1 h with the Erk1/Erk2 inhibitor PD98059 (PD, 10 μM), the p38 MAPK inhibitor SB203580 (SB, 10 μM), or the JNK inhibitor SP600125 (SP, 10 μM) and the vehicle control dimethyl sulfoxide (DMSO) followed by the administration of TGF-β1 (2.5 ng/ml) for 48 h. The inhibitory effects of these MAPK inhibitors on MAPK pathways were evaluated by the protein expressions of downstream signaling pathways of MAPK using Rabbit polyclonal anti-human antibodies for phospho-p44/42 MAPK(Erk1/2)(Thr202/Tyr204), phospho-MAPKAPK-2(Thr334), MAPKAPK-2, phosphor-c-Jun(Ser73) and c-Jun.
(TIF)

S3 Fig The effects of knock-down of the expressions of MAPK1 or MAPK8 on TGF-β1-induced IGFBP7. We transfected HRPTECs with control, MAPK1- or MAPK8-specific siRNAs (25 nM final concentrations) and then stimulated the cells with TGF-β1 (2.5 ng/ml, 48 h). Lowering the expressions of MAPK1 or MAPK8 proteins did not affect the TGF-β1-induced IGFBP7 expression.
(TIF)

S1 Table The sequences of the siRNAs for knock-down of targeted genes. The sequences of the siRNAs of ON-TARGET plus are available from Dharmacon. All siRNAs used in the manuscript including IGFBP7 siRNA (Santa Cruz Biotechnology) are multiple.
(DOCX)
