# Supplementary figures and images for: Role of IGFBP7 in Diabetic Nephropathy: TGF-β1 Induces IGFBP7 via Smad2/4 in Human Renal Proximal Tubular Epithelial Cells
Source: PLoS One. 2016 Mar 14;11(3):e0150897. doi: 10.1371/journal.pone.0150897 (PMC4790858; doi:10.1371/journal.pone.0150897)

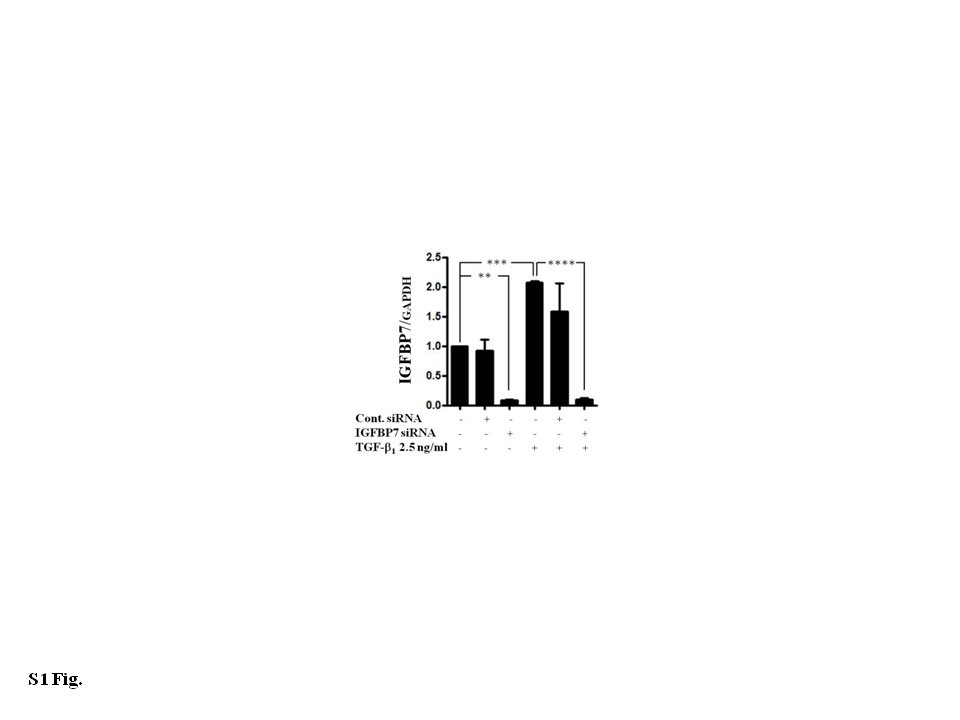

Supplement: S1 Fig — Forty-eight hours after HRPTECs were transfected with IGFBP7 siRNAs, HRPTECs were serum-starved for an additional 24 h and subsequently stimulated with TGF-β1 (2.5 ng/ml) for 48 h. The efiiciency of knock-down of IGFBP7 by specific IGFBP7 siRNAs was evaluated by qRT-PCR. TGF-β1 significantly induced IGFBP7 mRNA (2.075±0.02, p<0.001). IGFBP7 siRNAs decreased IGFBP7 mRNA expression under 10% of control (without TGF-β1, 0.084±0.02, p<0.001, with TGF-β1, 0.096±0.02, p<0.0001). Three separate experiments were performed per protocol, and each treatment group was assayed in duplicate. Values shown represent means ± SD. We performed an analysis of variance (ANOVA) and used post hoc Bonferroni tests. p**<0.01, p***<0.001, p****<0.0001. (TIF) [file pone.0150897.s001.tif]

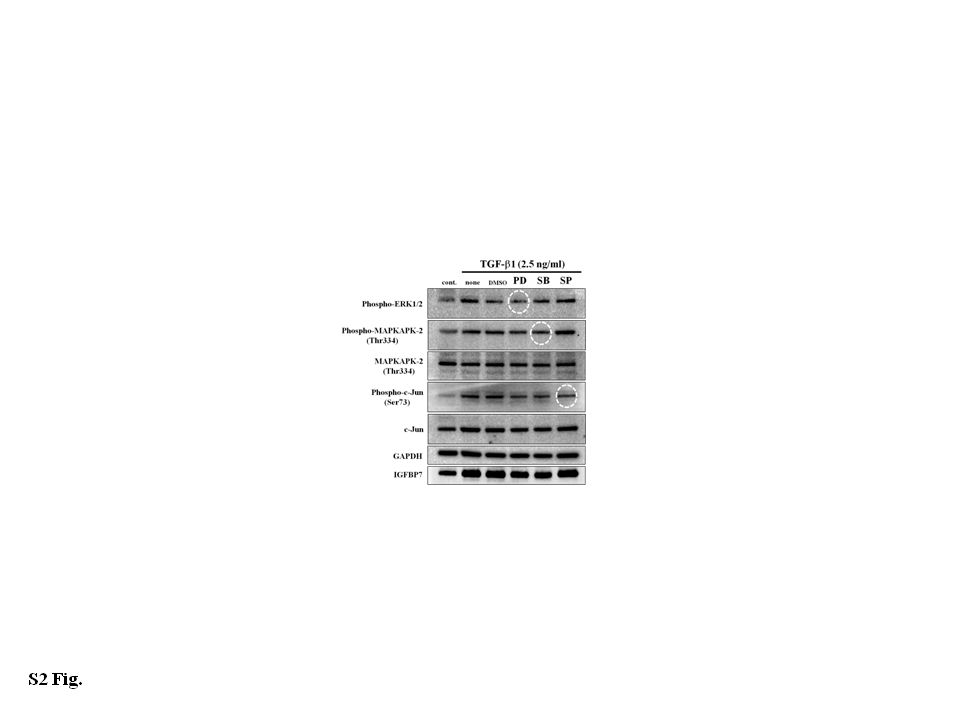

Supplement: S2 Fig — HRPTECs were treated for 1 h with the Erk1/Erk2 inhibitor PD98059 (PD, 10 μM), the p38 MAPK inhibitor SB203580 (SB, 10 μM), or the JNK inhibitor SP600125 (SP, 10 μM) and the vehicle control dimethyl sulfoxide (DMSO) followed by the administration of TGF-β1 (2.5 ng/ml) for 48 h. The inhibitory effects of these MAPK inhibitors on MAPK pathways were evaluated by the protein expressions of downstream signaling pathways of MAPK using Rabbit polyclonal anti-human antibodies for phospho-p44/42 MAPK(Erk1/2)(Thr202/Tyr204), phospho-MAPKAPK-2(Thr334), MAPKAPK-2, phosphor-c-Jun(Ser73) and c-Jun. (TIF) [file pone.0150897.s002.tif]

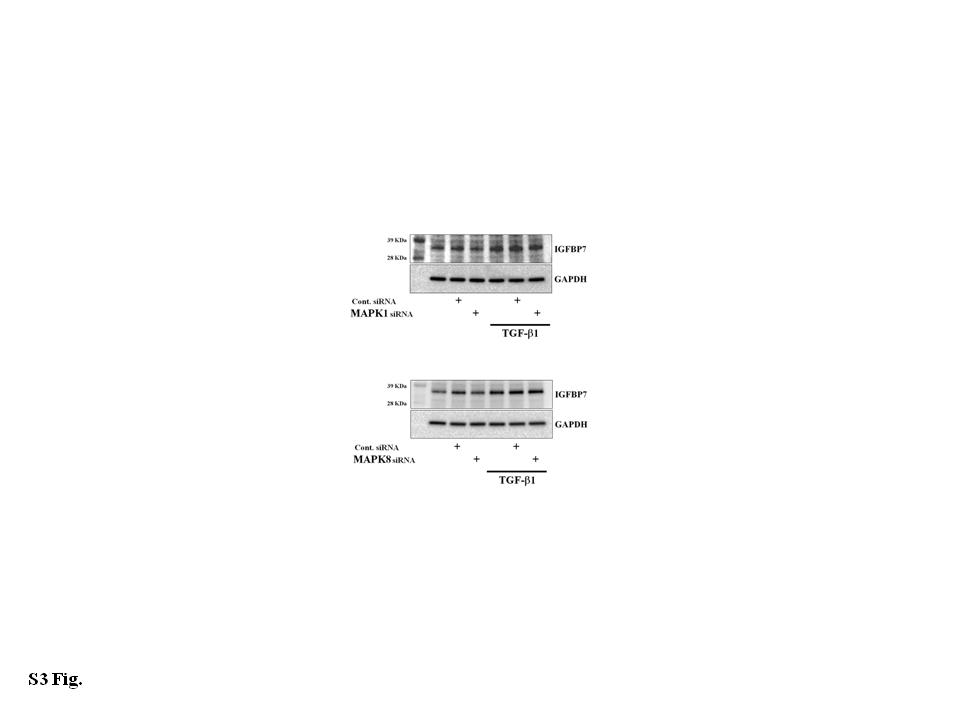

Supplement: S3 Fig — We transfected HRPTECs with control, MAPK1- or MAPK8-specific siRNAs (25 nM final concentrations) and then stimulated the cells with TGF-β1 (2.5 ng/ml, 48 h). Lowering the expressions of MAPK1 or MAPK8 proteins did not affect the TGF-β1-induced IGFBP7 expression. (TIF) [file pone.0150897.s003.tif]
