# Supplementary material for: Role of IGFBP7 in Diabetic Nephropathy: TGF-β1 Induces IGFBP7 via Smad2/4 in Human Renal Proximal Tubular Epithelial Cells
Source: PLoS One. 2016 Mar 14;11(3):e0150897. doi: 10.1371/journal.pone.0150897 (PMC4790858; doi:10.1371/journal.pone.0150897)
Supplement: S1 Table — The sequences of the siRNAs of ON-TARGET plus are available from Dharmacon. All siRNAs used in the manuscript including IGFBP7 siRNA (Santa Cruz Biotechnology) are multiple. (DOCX) [file pone.0150897.s004.docx]

**S1 Table. The sequences of the siRNAs for knock-down of targeted genes.**

| Target gene | The sequence of the siRNAs |
| --- | --- |
| **Human SMAD2** (L-003561) | GAAUUGAGCCACAGAGUAA |
|  | GGUUUACUCUCCAAUGUUA |
|  | UCAUAAAGCUUCACCAAUC |
|  | ACUAGAAUGUGCACCAUAA |
| **Human SMAD3** (L-020067) | CAACAGGAAUGCAGCAGUG |
|  | GAGUUCGCCUUCAAUAUGA |
|  | GGACGCAGGUUCUCCAAAC |
|  | UUAGAGACAUCAAGUAUGG |
| **Human SMAD4** (L-003902) | GCAAUUGAAAGUUUGGUAA |
|  | CCCACAACCUUUAGACUGA |
|  | GAAUCCAUAUCACUACGAA |
|  | GUACAGAGUUACUACUUAG |
| **Human MAPK1** Also known as **ERK; p38** (L-003555) | UCGAGUAGCUAUCAAGAAA |
|  | CACCAACCAUCGAGCAAAU |
|  | GGUGUGCUCUGCUUAUGAU |
|  | ACACCAACCUCUCGUACAU |
| **Human MAPK8** Also known as **JNK**  (L-003514) | GCCCAGUAAUAUAGUAGUA |
|  | GGCAUGGGCUACAAGGAAA |
|  | GAAUAGUAUGCGCAGCUUA |
|  | GAUGACGCCUUAUGUAGUG |
| Non-targeting (D-001810) | UGGUUUACAUGUCGACUAA |
|  | UGGUUUACAUGUUGUCUGA |
|  | UGGUUUACAUGUUUUCUGA |
|  | UGGUUUACAUGUUUUCCUA |
